# Leptospira gorisiae sp. nov, L. cinconiae sp. nov, L. mgodei sp. nov, L. milleri sp. nov and L. iowaensis sp. nov: five new species isolated from water sources in the Midwestern United States

**DOI:** 10.1099/ijsem.0.006595

**Published:** 2025-01-07

**Authors:** Camila Hamond, Bienvenido Tibbs-Cortes, Luis G. V. Fernandes, Karen LeCount, Ellie J. Putz, Tammy Anderson, Patrick Camp, Tod Stuber, Jessica Hicks, Hans van der Linden, Priscyla dos Santos Ribeiro, Darrell O. Bayles, Linda K. Schlater, Jarlath E. Nally

**Affiliations:** 1 National Veterinary Services Laboratories, Animal and Plant Health Inspection Service, U.S. Department of Agriculture, Ames, IA, USA; 2 Infectious Bacterial Diseases Research Unit, Agricultural Research Service, U.S. Department of Agriculture, Ames, IA, USA; 3 Department of Medical Microbiology and Infection Prevention, World Organisation for Animal Health and National Collaborating Centre for Reference and Research on Leptospirosis, Amsterdam University Medical Center, University of Amsterdam, Amsterdam, Netherlands

**Keywords:** *Leptospira*, leptospirosis, pathogen, saprophyte, water source, whole-genome sequencing

## Abstract

Isolates of *Leptospira* spp. were cultured from water sources at five different sites in central Iowa in the Midwestern United States and characterized by whole-genome sequencing. Isolates were helix-shaped and motile. Genome sequence analyses determined that the isolates could be clearly distinguished from other species described in the genus *Leptospir*a and included one species that belonged to the pathogen subclade P1, one species that belonged to the pathogen subclade P2 and three species that belonged to the saprophyte subclade S1. The names *Leptospira gorisiae* sp. nov. (type strain WS92.C1^T^=NVSL-WS92.C1^T^=KIT0303^T^), *Leptospira cinconiae* sp. nov. (type strain WS58.C1^T^=NVSL-WS58.C1^T^=KIT0304^T^), *Leptospira mgodei* sp. nov. (type strain WS4.C2^T^=NVSL.WS4.C2^T^=KIT0305^T^), *Leptospira iowaensis* sp. nov. (type strain WS39.C2^T^=NVSL-WS39.C2^T^=KIT0306^T^) and *Leptospira milleri* sp. nov. (type strain WS60.C2^T^=NVSL-WS60.C2^T^=KIT0307^T^) are proposed.

## Introduction

Leptospirosis is a global zoonotic disease that causes significant morbidity and mortality in human and animal populations [1]. Animal leptospirosis can significantly impact production on farms due to infertility, abortions, stillbirths, weak offspring and decreased milk production [2]. The most common way that humans or animals contract leptospirosis is by exposure to soil or water contaminated with the urine of reservoir animals infected with pathogenic *Leptospira* [1,3].

The genus *Leptospira* is diverse and divided into 69 species [4,8]. Phylogenomic analysis separates the genus into two clades: the S clade that contains noninfective saprophytes isolated from the environment and the P clade that contains pathogens/intermediates responsible for infections in humans and animals. Each S and P clade is further subdivided into two subclades: subclades S1 and S2 contain 23 and 5 species, respectively, while subclades P1 and P2 contain 20 and 21 species, respectively. More recently, three new species belonging to subclade S1 were isolated from environmental biofilms [9].

The discovery of novel species of *Leptospira*, particularly species belonging to the pathogen subclades P1 and P2, is critical for the development of efficacious detection and diagnostic tools [4]. Here, we describe five new species of *Leptospira* that were isolated from water sources in central Iowa, the analyses of which indicate that two new species are predicted to be pathogenic and three new species are saprophytes.

## Isolation

*Leptospira* strains WS4, WS39, WS58, WS60 and WS92 were originally isolated from water sources collected in Iowa, United States, as previously described [3]. In brief, a 250 µl aliquot of each water sample was inoculated at the time of collection into 5 ml of HAN (Hornsby–Alt–Nally) liquid media [10]. In the laboratory, 500 µl of the inoculated media was then used to inoculate 2×5 ml of HAN media containing STAFF (sulfamethoxazole, 40 µg ml^−1^; trimethoprim, 20 µg ml^−1^; amphotericin B, 5 µg ml^−1^; fosfomycin, 400 µg ml^−1^; and 5-fluorouracil, 100 µg ml^−1^) [11] and incubated at 29 °C. Positive cultures were characterized by whole-genome sequencing. To generate clonal isolates of type strains in accordance with guidelines from the Subcommittee on the Taxonomy of Leptospiraceae and to ensure that type strains for new species represent one single species of *Leptospira*, cultures of each new species were propagated in HAN media at 29 and 37 °C, diluted to 10^4^ leptospires per ml, and 100 µl then used to inoculate separate HAN media agar plates that were also incubated at 29 and 37 °C. Individual colonies were detectable on agar plates by 10 days (Supplementary Fig. S1, available in the online Supplementary Material) and single colonies were selected to act as type strains. Type strains designated WS4.C2^T^, WS39.C2^T^ and WS92.C1^T^ were selected from agar plates incubated at 29 °C, and type strains designated WS58.C1^T^ and WS60.C2^T^ were selected from agar plates incubated at 37 °C.

## Phenotype

**Fig. 1.**
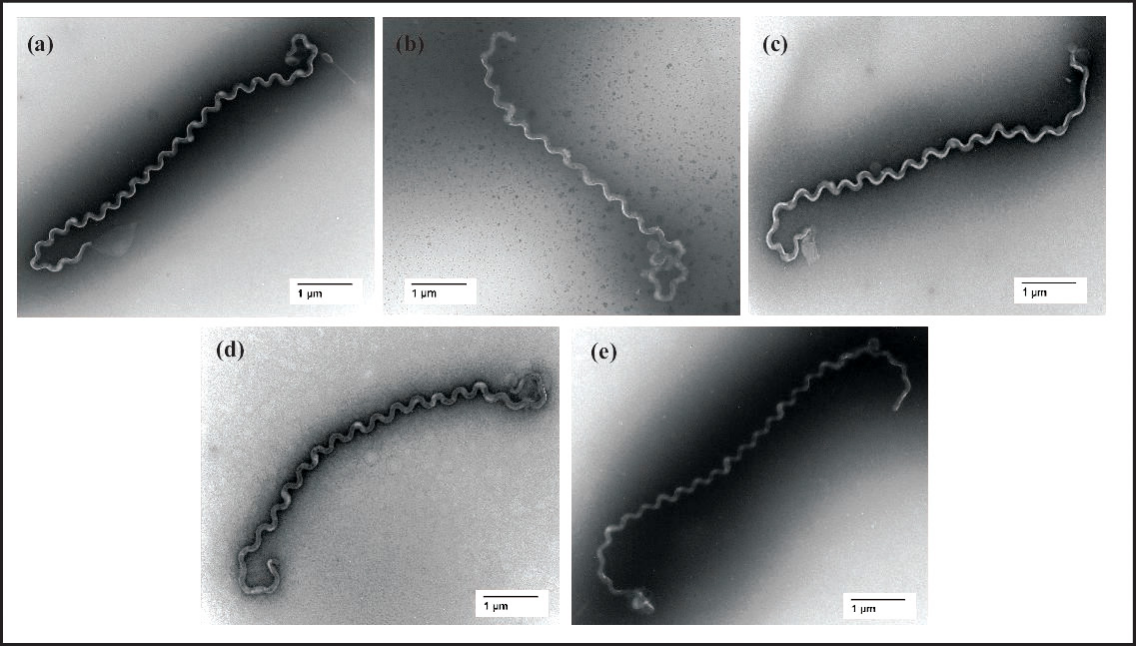
Representative images of new species of *Leptospira* by transmission electron microscopy. (**a**) *Leptospira mgodei* sp. nov., (**b**) *Leptospira iowaensis* sp. nov., (**c**) *Leptospira cinconiae* sp. nov., (**d**) *Leptospira milleri* sp. nov. and (e) *Leptospira gorisiae* sp. nov.

Strains WS4, WS39, WS58, WS60 and WS92 presented with similar morphologies and motility to that of other members of the genus *Leptospira* when visualized by transmission electron microscopy (Fig. 1) and dark-field microscopy. Representative cells (*N*=10) of strain WS4 were 12.35±2.91 µm long, 0.16 µm in diameter, with a wavelength of ~0.47 µm (Fig. 1a). Cells (*N*=10) of WS39 were 14.9±2.08 µm long, 0.17 µm in diameter, with a wavelength of ~0.64 µm (Fig. 1b). Cells (*N*=10) of WS58 were 16.15±1.18 µm long, 0.15 µm in diameter, with a wavelength of ~0.71 µm (Fig. 1c). Cells (*N*=10) of WS60 were 18.5±1.2 µm long, 0.18 µm in diameter, with a wavelength of ~0.71 µm (Fig. 1d). Cells (*N*=10) of WS92 were 14.42±4.34 µm long, 0.12 µm in diameter, with a wavelength of ~0.55 µm (Fig. 1e). Strains WS4, WS39 and WS92 grew better on HAN agar plates incubated at 29 °C, while strains WS58 and WS60 grew better on HAN agar plates incubated at 37 °C (Supplementary Fig. S1).

**Fig. 2.**
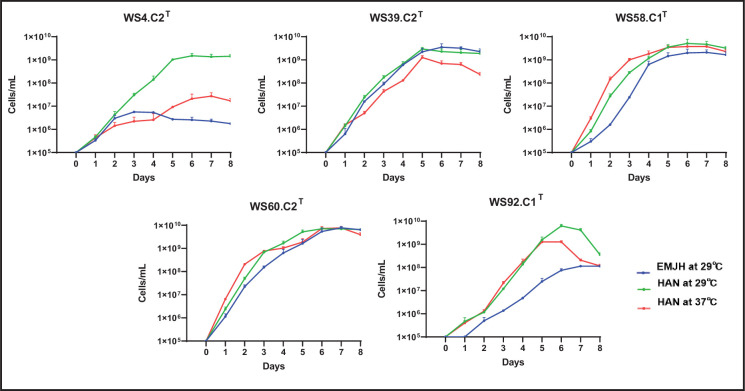
Growth curves of type strains *Leptospira mgodei* strain WS4.C2^T^ (clade S1), *Leptospira iowaensis* strain WS39.C2^T^ (clade S1), *Leptospira cinconiae* strain WS58C.1^T^ (clade P2), *Leptospira millerii* strain WS60.C2^T^ (clade S1) and *Leptospira gorisiae* WS92.C1^T^ (clade P1). Leptospires were inoculated in EMJH and HAN media at the conventional temperature of 29 °C, and in HAN media at 37 °C, in triplicate, at an initial concentration of 10^5^ cells per ml and counted daily by dark-field microscopy.

All type strains selected from single colonies on HAN agar plates grew well (>10^8^ leptospires per ml) in HAN liquid media incubated at 29 °C (Fig. 2). Strains WS39.C2^T^, WS58.C1^T^, WS60.C2^T^ and WS92.C1^T^ also grew well (>10^8^ leptospires per ml) in HAN media incubated at 37 °C and EMJH media incubated at 29 °C. However, strain WS4.C2^T^ grew relatively poorly in HAN media incubated at 37 °C and EMJH media incubated at 29 °C (Fig. 2).

## Proteins and LPS

**Fig. 3.**
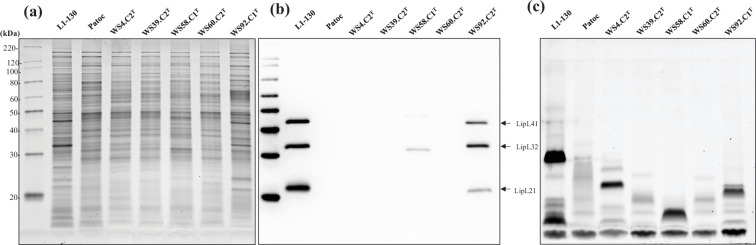
Representative images showing total protein profile, (**a**) immunoblotting with anti-LipL32, anti-LipL21 and anti-LipL41, (**b**) and total LPS profiles. (**c**) About 5 µg of each strain was loaded per lane. Molecular mass markers are indicated.

Leptospires (mid-late log phase, 1–3×10^8^ leptospires per ml) were harvested by centrifugation (10 000×*g*, 4 °C, 30 min), washed twice with PBS and processed for one-dimensional (1-D) sodium dodecyl-sulphate polyacrylamide gel electrophoresis on 12% acrylamide gels (BioRad, Hercules, CA, USA) as per manufacturer’s guidelines. Proteins were visualized with Sypro Ruby (Invitrogen) and LPS was visualized with Pro-Q Emerald 300 (Invitrogen) as per manufacturer’s guidelines. For immunoblotting, samples were transferred by semi-dry transfer (Amersham TE77 PWR) to Immobilon-P transfer membranes (Millipore, 220 Bedford, MA, USA) and blocked overnight at 4 °C with Starting Block (PBS) blocking buffer (Thermo Fisher). Membranes were individually incubated with indicated antisera diluted in blocking buffer (anti-LipL32 at 1 : 4000, anti-LipL21 at 1 : 2000 and anti-LipL41 at 1 : 4000) [12,13], followed by incubation with horseradish-peroxidase anti-rabbit immunoglobulin G conjugate (Sigma, St. Louis, MO, USA) diluted 1 : 4000 in blocking buffer. Bound conjugates were detected using Clarity Western ECL substrate (BioRad) and images were acquired using a Bio-Rad ChemiDoc MP imaging system. Strains WS4.C2^T^, WS39.C2^T^ and WS60.C2^T^ have similar protein profiles to that of the saprophyte *L. biflexa* serovar Patoc (Fig. 3a), while strain WS92.C1^T^ has a similar protein profile to that of the pathogen *L. interrogans* serovar Copenhageni strain L1-130 (Fig. 3a). Strains WS58.C1^T^ and WS92.C1^T^ express the outer membrane lipoprotein LipL32 that is conserved among pathogenic species of *Leptospira* (Fig. 3b). The pathogen-associated outer membrane proteins LipL41 and LipL21 are also expressed by strain WS92.C1^T^ (Fig. 3b). Each new species also presents with different LPS profiles (Fig. 3c).

## Genome features

Closed genomes were generated for all type strains. DNA was extracted from 5 ml of culture using the Maxwell RSC Purefood Purification Pathogen kit (Promega Corporation, Madison, WI, USA) for Illumina Genome Sequencing or the Nanobind CBB Big DNA Kit (Circulomics, Baltimore, MD, USA) for Nanopore Genome Sequencing. Samples were prepared for sequencing on the Illumina MiSeq platform using the Illumina DNA prep sample method and sequenced using a 2×250 v2 paired-end chemistry cartridge per the manufacturer’s instructions. Prior to Nanopore sequencing, the PacBio Short Read Eliminator Kit XS was used to select fragments longer than 10 kb, following the manufacturer’s instructions. The size-selected DNA was indexed and barcoded using the Oxford Nanopore Native Barcoding Kit 24 V14 (SQK-NBD114.24) following the manufacturer’s instructions. Samples were pooled and loaded onto a Nanopore flow cell FLO-MIN114 and run for 72 h.

BBDuk was used to trim residual sequencing adapters from raw Illumina reads and to perform quality trimming of both ends of each read to a PHRED quality score of 30. After trimming, reads with an average PHRED quality score of <30 and a length <150 bp were discarded [14]. Long reads less than 1000 bp were discarded using Filtlong, and subsequently the worst 5% of reads in terms of quality score were also removed from the dataset [15]. Trycycler v0.5.5 was used to generate a high-quality hybrid assembly for each new species following the vignette at https://github.com/rrwick/Trycycler/wiki/Generating-assemblies [16]. Briefly, for each species, 12 long-read subsets were generated. Flye, Minasm and Raven were each used to assemble four subsets each into draft contigs [17,19]. Draft contigs from the 12 assembled subsets were then clustered and visually inspected, and contigs within the same cluster were circularized, oriented to the same start position and aligned against each other using the Trycycler ‘reconcile’ command. Contigs that failed to circularize or align were removed from the analysis. In total, 2 contigs from WS4, 5 contigs from WS39, 2 contigs from WS58, 5 contigs from WS60 and 3 contigs from WS92 were removed. The initial (not subset) cleaned long reads were then aligned against the contig clusters and used to generate consensus long read assemblies. These were then polished with the cleaned Illumina data using Polypolish to generate high-quality genomes for each species [20]. The final genomes were subsequently annotated with Prokka. Using the rotate package, contigs representing chromosome I were reoriented to begin with *dnaA*, while contigs representing chromosome II were reoriented to begin with *soJ* or *parA* [21]. Genomes were submitted to NCBI for deposition and final annotation via the NCBI prokaryotic genome annotation pipeline [22,24].

The minimum coverage for any genome was 229× (WS39.C2^T^), and all contigs were circularized and closed, yielding complete chromosomes and plasmids. Closed genomes ranged in size from 3 873 836 nt in the case of WS60.C2^T^ to 4 527 809 in the case of WS92.C1^T^. In addition to chromosomes I and II, WS4.C2^T^, WS39.C2^T^and WS92.C1^T^ each possessed one plasmid, and WS60.C2^T^ was found to harbour two plasmids. WS60.C2^T^ also had both the largest and smallest plasmids with sizes of 113 167 nt and 55 184 nt, respectively. Total annotated CDSs per genome ranged from 3585 (WS60.C2^T^) to 4059 (WS92.C1^T^) (Table 1).

**Table 1.** Genome features for *Leptospira mgodei* strain WS4.C2^T^, *Leptospira iowaensis* strain WS39.C2^T^, *Leptospira cinconiae* strain WS58.C1^T^, *Leptospira milleri* strain WS60.C2^T^ and *Leptospira gorisiae* strain WS92.C1^T^

**Species**	Strain	Assembly size (nt)	Chr 1(nt)	Chr II(nt)	Plasmid I(nt)	Plasmid II(nt)	Coverage	G+C content	No. of CDS
*L. mgodei*	WS4.C2^T^	4,041,414	3,711,287	293,914	66,213	na	685×	39.00 mol%	3764
*L. iowaensis*	WS39.C2^T^	4,065,238	3,720,064	273,036	72,138	na	229×	37.00 mol%	3823
*L. cinconiae*	WS58.C1^T^	4,092,442	3,719,482	372,960	na	na	407×	41.76 mol%	3756
*L. milleri*	WS60.C2^T^	3,873,836	3,436,525	268,960	113,167	55,184	342×	38.70 mol%	3585
*L. gorisiae*	WS92.C1^T^	4,527,809	4,056,702	407,834	63,273	na	429×	41.50 mol%	4059

## Genome phylogenetics

Genome analyses, as indicated below, indicated that each type strain represents a new species, which were named as follows: WS4.C2^T^ = *L. mgodei*, WS39.C2^T^ = *L. iowaensis*, WS58.C1^T^ = *L. cinconiae*, WS60.C2^T^ = *L. milleri*, WS92.C1^T^ = *L. gorisiae*.

**Fig. 4.**
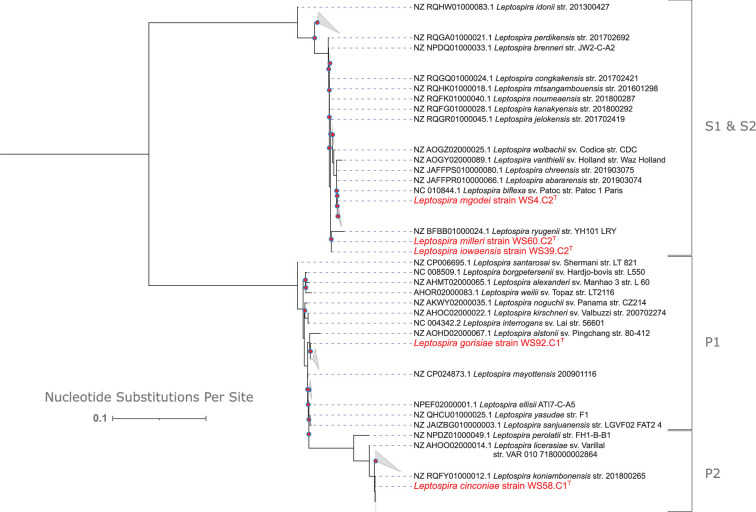
An abbreviated version of the 16S rRNA gene-based consensus tree displaying the phylogenetic relationships between reference *Leptospira* species (black text labels) and the five novel species introduced in this work (red text labels). The tree was visualized using the Interactive Tree of Life, and some clades are collapsed for brevity (grey triangles). The tree is rooted at the midpoint and has a log-likelihood of −4844. Totally, 1000 bootstrap replicates were performed for consensus tree construction, and nodes with bootstrap values below 70% are indicated with a red circle.

For phylogenetic analysis based on the 16S rRNA gene, the full 16S rRNA gene sequence was extracted from the genome of each of the 5 new species as well as the 69 reference genomes of all *Leptospira* species (Supplementary Table S1, downloaded from https://leptosociety.org/resources/ on 20 June 2024) using Barrnap [25]. The resultant sequences were aligned using MAFFT v7.505 with the l-INS-I method with 1000 iterative refinements [26,27]. This generated an alignment file that was then used for phylogenetic tree construction via IQ-TREE [28]. The ModelFinder method of IQ-TREE was enabled and identified TN+F+I+R2 as the best-fit model [29]. Bootstrap approximation using UFBOOT was performed with 1000 replicates, and the -bnni option was enabled to mitigate severe violations of the chosen model during bootstrapping [30]. The resulting tree was visualized using the Interactive Tree of Life v5 [31]. For easier visualization, the tree is presented rooted at the midpoint with some clades collapsed in Fig. 4; the same tree unrooted with all taxa visible is provided in Supplementary Fig. S2. Based on 16S rRNA gene phylogeny, *L. cinconiae* and *L. gorisiae* fall within the pathogenic subclades P2 and P1, respectively. The remaining novel species are shown to belong to the saprophytic clade, but 16S rRNA gene phylogeny is not capable of delineating between members of the S1 and S2 subclades (Figs 4 and Supplementary Fig. S2).

**Fig. 5.**
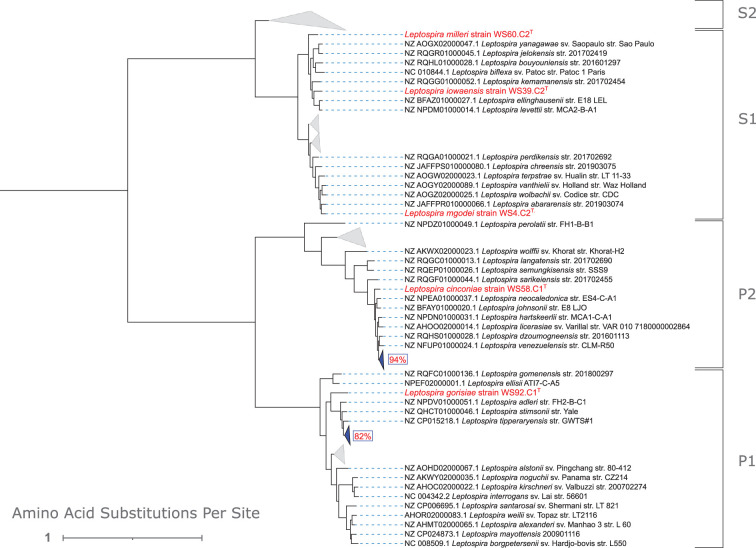
An abbreviated version of the core genome consensus tree (based on 714 protein-coding sequences) displaying the phylogenetic relationship between reference *Leptospira* species (black text labels) and the five novel species introduced in this work (red text labels). The tree was visualized using the Interactive Tree of Life, and some clades are collapsed for brevity (grey triangles). The tree is rooted at the midpoint and has a log-likelihood of −3 834 073. Totally 1000 bootstrap replicates were performed for consensus tree construction, and clades with bootstrap values below 95% are collapsed (blue triangles, blue box with red text indicates bootstrap value).

For phylogenomic analysis, PanACoTA v1.3.1 was utilized to identify a core genome among all 74 (novel and reference) *Leptospira* species [32]. The thresholds for MASH distance, maximum L90 of a genome and maximum number of contigs per genome were adjusted to ensure that all 74 genomes were kept during the ‘prepare’ and ‘annotate’ modules of PanACoTA. All annotated protein-coding sequences across genomes were next grouped into families by the ‘pangenome’ module based on a minimum amino acid similarity threshold of 50%; the number of members of each protein family in each genome was subsequently quantified. From this information, a strict persistent genome was generated consisting of the 714 protein families that were represented by a single homolog in each of the 74 genomes. The homologs within each family were subsequently aligned using MAFFT within PanACoTA. Alignments were trimmed to retain parsimony informative sites and remove gap sites using the ‘kpi-smart-gap’ method of ClipKIT v2.3.0 [33]. PhyKIT v1.19.9 was used to concatenate all 714 trimmed alignments and generate a partition file [34]; these were then used as input for phylogenetic tree construction via IQ-TREE v2.3.0 [28,35]. Within IQ-TREE, ModelFinder was used to identify the optimal edge-proportional partition model prior to tree reconstruction with UFBOOT using 1000 bootstraps. As with the 16S rRNA gene phylogeny, the tree was visualized in the Interactive Tree of Life, and abbreviated and complete versions of the tree are provided (Figs 5 and Supplementary Fig. S3, respectively). The phylogenetic location of the five novel species as determined by the phylogenomic method agrees with the 16S rRNA gene phylogeny, and the increased resolution of the phylogenomic method demonstrates that the three saprophytic species belong to the S1 subclade (Figs 4 and Supplementary Fig. S2).

**Fig. 6.**
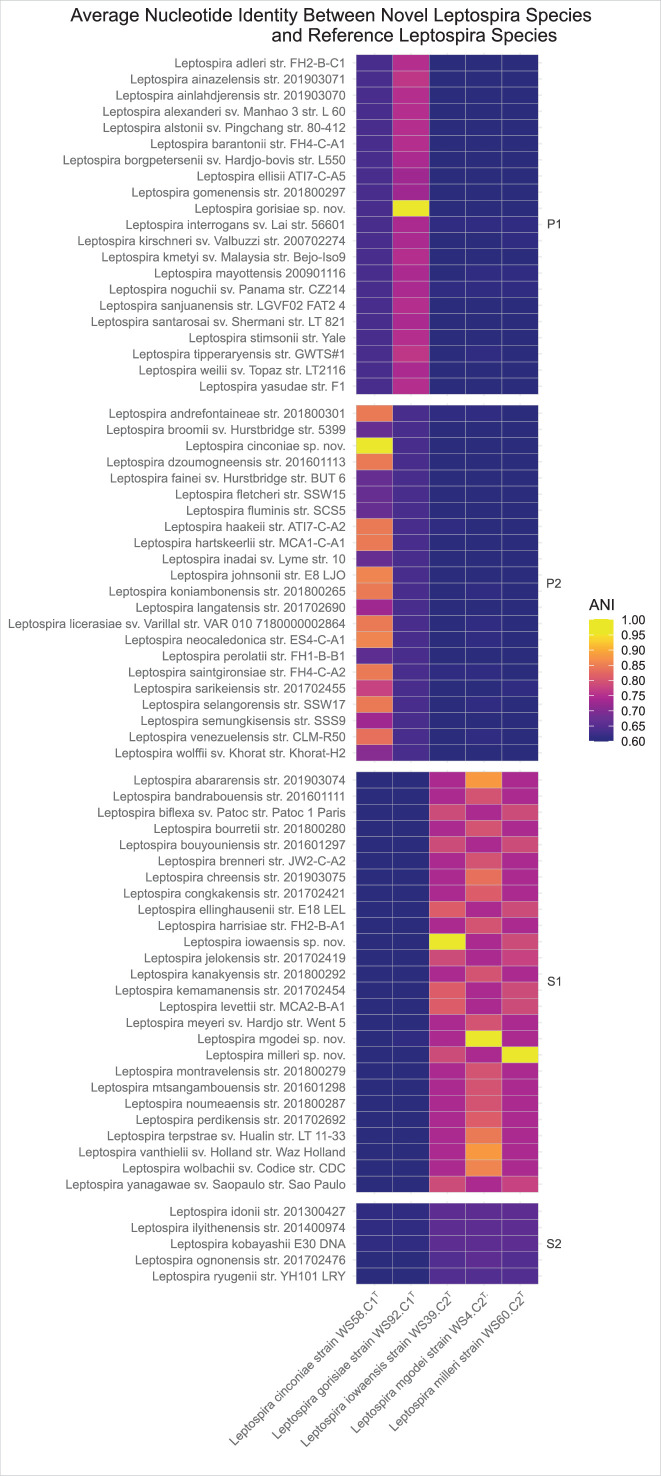
Heatmap displaying the ANI (as calculated by OrthoANI) between the genomes of the five novel species introduced in this work and the existing reference *Leptospira* species. Numeric ANI values calculated by OrthoANI used to generate Fig. 6 are provided in Supplementary Table S2.

The average nucleotide identity (ANI) for each of the five new species against reference genomes for each species (Supplementary Table S1) was calculated using the OrthoANI algorithm [36] implemented in Python [37] and all were well below the 95% threshold recommended for new species delineation (Fig. 6 and Supplementary Table S2) [38,39]. For *L. gorisiae* strain WS92.C1^T^, the closest ANI was 79% for *L. tipperaryensis*. For *L. cinconiae* strain WS58.C1^T^, the closest ANI was 87% for *L. johnsonii*. For *L. mgodei* strain WS4.C2T, the closest ANI was 89% for *L. abararensis*. For * L. iowaensis* strain WS39.C2^T^, the closest ANI was 83% for *L. ellinghausen*. For *L. milleri* strain WS60.C2^T^, the closest ANI was 81% for *L. ellinghausen*.

## Serotyping

Strains WS4.C2^T^, WS39.C2^T^, WS58.C1^T^, WS60.C2^T^ and WS92.C1^T^ were serotyped using the MAT method with a panel of 48 polyclonal rabbit reference antisera representing over 29 serogroups and *Leptonema* (Supplementary Table S3) [40]. No significant titres were detected for strains WS4.C2^T^, WS39.C2^T^, WS58.C1^T^ or WS92.C1^T^, representing subclades S1, S1, P2 and P1, respectively. Strain WS60.C2^T^ of subclade S1 had a titre of 1 : 1280 for serogroup Tarassovi, but additional testing with monoclonal antibodies was negative for any serovar within this serogroup.

## Description of *Leptospira mgodei* sp. nov.

*Leptospira mgodei* (mgo’de.i. N.L. gen. n. *mgodei*, named in honour of Georgies Mgode, a Tanzanian leptospirologist who made significant contributions to the study of human and animal leptospirosis) (Subclade S1).

Cells are 12.35±2.91 µm long and 0.16 µm in diameter, presenting with a wavelength of ~0.47 µm; cells are thin and helical-shaped, with hooked and/or spiral ends to which flagella are inserted. Cells are highly motile and grow better in HAN media at 29 °C compared to EMJH media at 29 °C or HAN media at 37 °C. When cells are seeded onto HAN solid media and incubated at 29 and 37 °C, colonies can be observed at 10 days only at 29 °C . The genomic G+C content of the type strain WS4.C2^T^ (=NVSL-WS4.C2 ^T^=KIT0305^T^) is 39.00 mol%.

## Description of *Leptospira iowaensis* sp. nov.

*Leptospira iowaensis* (i.o.wa.en’sis. N.L. fem. adj. *iowaensis*, of Iowa, named after the state from which the isolate was recovered) (Subclade S1).

Cells are 14.9±2.08 µm long and 0.17 µm in diameter, presenting with a wavelength of ~0.64 µm; cells are thin and helical-shaped, with hooked and/or spiral ends to which flagella are inserted. Cells are highly motile and display vigorous growth at 29 °C (EMJH and HAN) and at 37 °C (HAN media). When cells are seeded onto HAN solid media and incubated at 29 and 37 °C, colonies can be observed at 10 days only at 29 °C. The genomic G+C content of the type strain WS39.C2^T^ (=NVSL-WS39.C2 ^T^=KIT0306^T^) is 37.00 mol%.

## Description of *Leptospira cinconiae* sp. nov.

*Leptospira cinconiae* (cin.co’ni.ae. N.L. gen. n. *cinconiae*, named in honour of Marina Cinco, an Italian leptospirologist who made significant contributions to the study of human and animal leptospirosis) (Subclade P2).

Cells are 16.15±1.18 µm long and 0.15 µm in diameter, presenting with a wavelength of ~0.71 µm; cells are thin and helical-shaped, with hooked and/or spiral ends to which flagella are inserted. Cells are highly motile and display vigorous growth at 29 °C in both EMJH and HAN media and at 37 °C in HAN media. When cells are seeded onto HAN solid media and incubated at 29 and 37 °C, colonies can be observed at 10 days. The genomic G+C content of the type strain WS58.C2^T^ (=NVSL-WS58.C1^T^=KIT0304^T^) is 41.76 mol%.

## Description of *Leptospira milleri* sp. nov.

*Leptospira milleri* (mil’le.ri. N.L. gen. n. *milleri*, named in honour of James N. Miller, an American spirochetologist who made significant contributions to the study of *Treponema*, *Leptospira* and *Borrelia*) (Subclade S1).

Cells are 18.5±1.2 µm long and 0.18 µm in diameter, presenting with a wavelength of ~0.71 µm; cells are thin and helical-shaped, with hooked and/or spiral ends to which flagella are inserted. Cells are highly motile and display vigorous growth at 29 °C in EMJH and HAN media and at 37 °C in HAN media. When cells are seeded onto HAN solid media and incubated at 29 and 37 °C, colonies can be observed at 10 days. The genomic G+C content of the type strain WS60.C2^T^ (=NVSL-WS60.C2 ^T^=KIT0307^T^) is 38.70 mol%.

## Description of *Leptospira gorisiae* sp. nov.

*Leptospira gorisiae* (go.ris’i.ae. N.L. gen. *gorisiae*, named in honour of Marga Goris, a Dutch leptospirologist who made significant contributions to the study of human and animal leptospirosis) (Subclade P1).

Cells are 14.42±4.34 µm long and 0.12 µm in diameter, presenting with a wavelength of ~0.55 µm; cells are thin and helical-shaped, with hooked and/or spiral ends to which flagella are inserted. Cells are highly motile and display vigorous growth at 29 °C in EMJH and HAN media and at 37 °C in HAN media. When cells are seeded onto HAN solid media and incubated at 29 and 37 °C, colonies can be observed at 10 days only at 29 °C. The genomic G+C content of the type strain WS92.C1^T^ (=NVSL-WS92.C1^T^=KIT0303^T^) is 41.50 mol%.

The GenBank accession numbers for 16S rRNA sequences for *L. cinconiae* strain WS58.C1^T^, *L. gorisiae* strain WS92.C1^T^, *L. iowaensis* strain WS39.C2^T^, *L. mgodei* strain WS4.C2^T^ and *L. milleri* strain WS60.C2^T^ are PQ066384, PQ066385, PQ066386, PQ066387 and PQ066388, respectively, and the GenBank accession numbers for their genomes are CP162137-CP162138, CP162130-CP162132, CP162142-CP162144, CP162139-CP162141 and CP162133-CP162136, respectively.

**16S GenBank Accession Numbers**:

**Table:** 

#Accession	Sequence ID
PQ066384	Leptospira_cinconiae_WS58.C1
PQ066385	Leptospira_gorisiae_WS92.C1
PQ066386	Leptospira_iowaensis_WS39.C2
PQ066387	Leptospira_mgodei_WS4.C2
PQ066388	Leptospira_milleri_WS60.C2

**Genome GenBank Accession Numbers**:

**Table:** 

Genome accession	Raw read accession	BioProject	BioSample.name
CP162130-CP162132	SAMN42533266	PRJNA1136487	Leptospira_gorisiae_WS92.C1
CP162133-CP162136	SAMN42533265	PRJNA1136487	Leptospira_milleri_WS60.C2
CP162137-CP162138	SAMN42533264	PRJNA1136487	Leptospira_cinconiae_WS58.C1
CP162139-CP162141	SAMN42533263	PRJNA1136487	Leptospira_mgodei_WS4.C2
CP162142-CP162144	SAMN42533262	PRJNA1136487	Leptospira_iowaensis_WS39.C2

## supplementary material

10.1099/ijsem.0.006595 Uncited Supplementary Material 1.

10.1099/ijsem.0.006595 Uncited Supplementary Material 2.

## Abbreviations

ANI: average nucleotide identity
CDS: coding DNA sequence
ECL: enhanced chemiluminesence
EMJH: Ellinghausen McCullough Johnson Harris
NCBI: National Center for Biotechnology Information
