# Supplementary material for: Leptospira gorisiae sp. nov, L. cinconiae sp. nov, L. mgodei sp. nov, L. milleri sp. nov and L. iowaensis sp. nov: five new species isolated from water sources in the Midwestern United States
Source: Int J Syst Evol Microbiol. 2025 Jan 7;75(1):006595. doi: 10.1099/ijsem.0.006595 (PMC11706286; doi:10.1099/ijsem.0.006595)
Supplement: Uncited Supplementary Material 1. [file ijsem-75-06595-s001.pdf]

Supplementary Figure 1

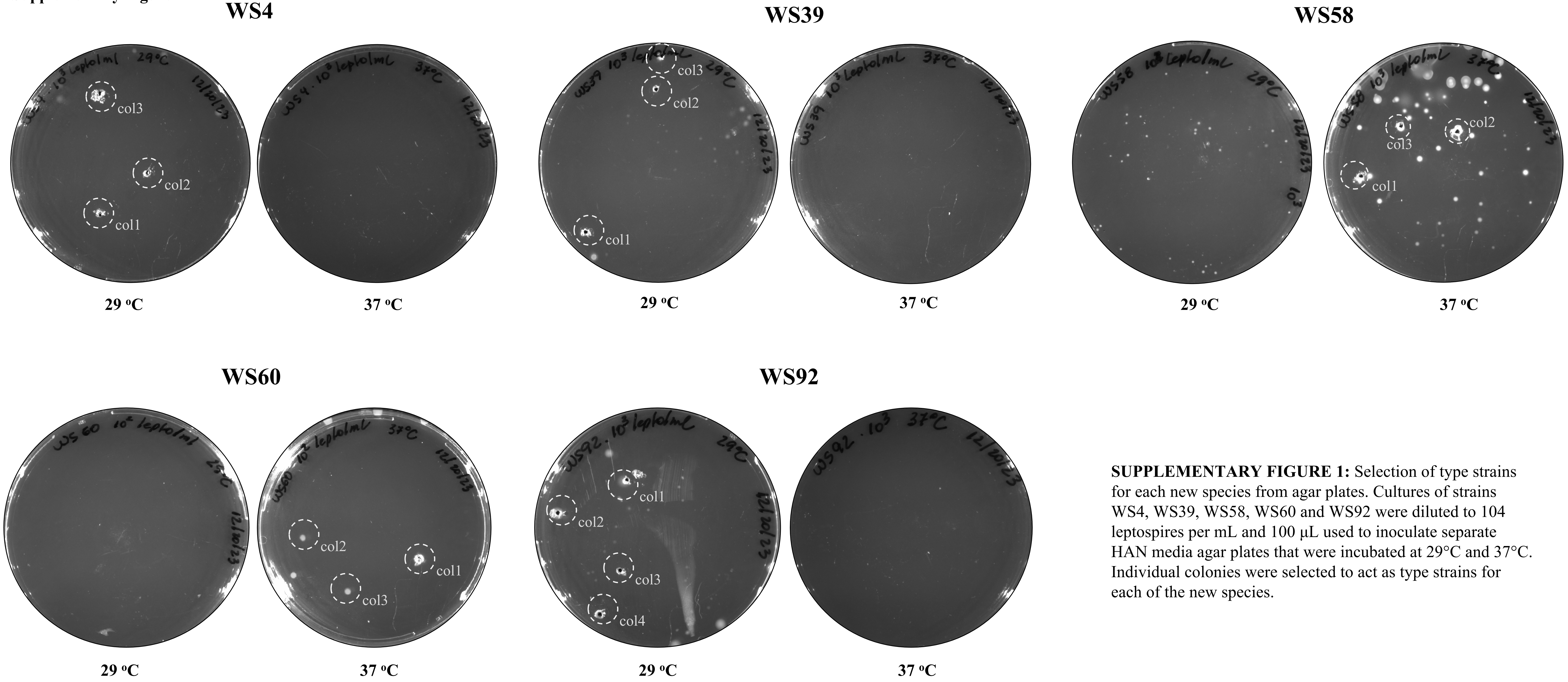

SUPPLEMENTARY FIGURE 1: Selection of type strains for each new species from agar plates. Cultures of strains WS4, WS39, WS58, WS60 and WS92 were diluted to 104 leptospires per mL and 100 µL used to inoculate separate HAN media agar plates that were incubated at 29°C and 37°C. Individual colonies were selected to act as type strains for each of the new species.

Supplementary Figure 2

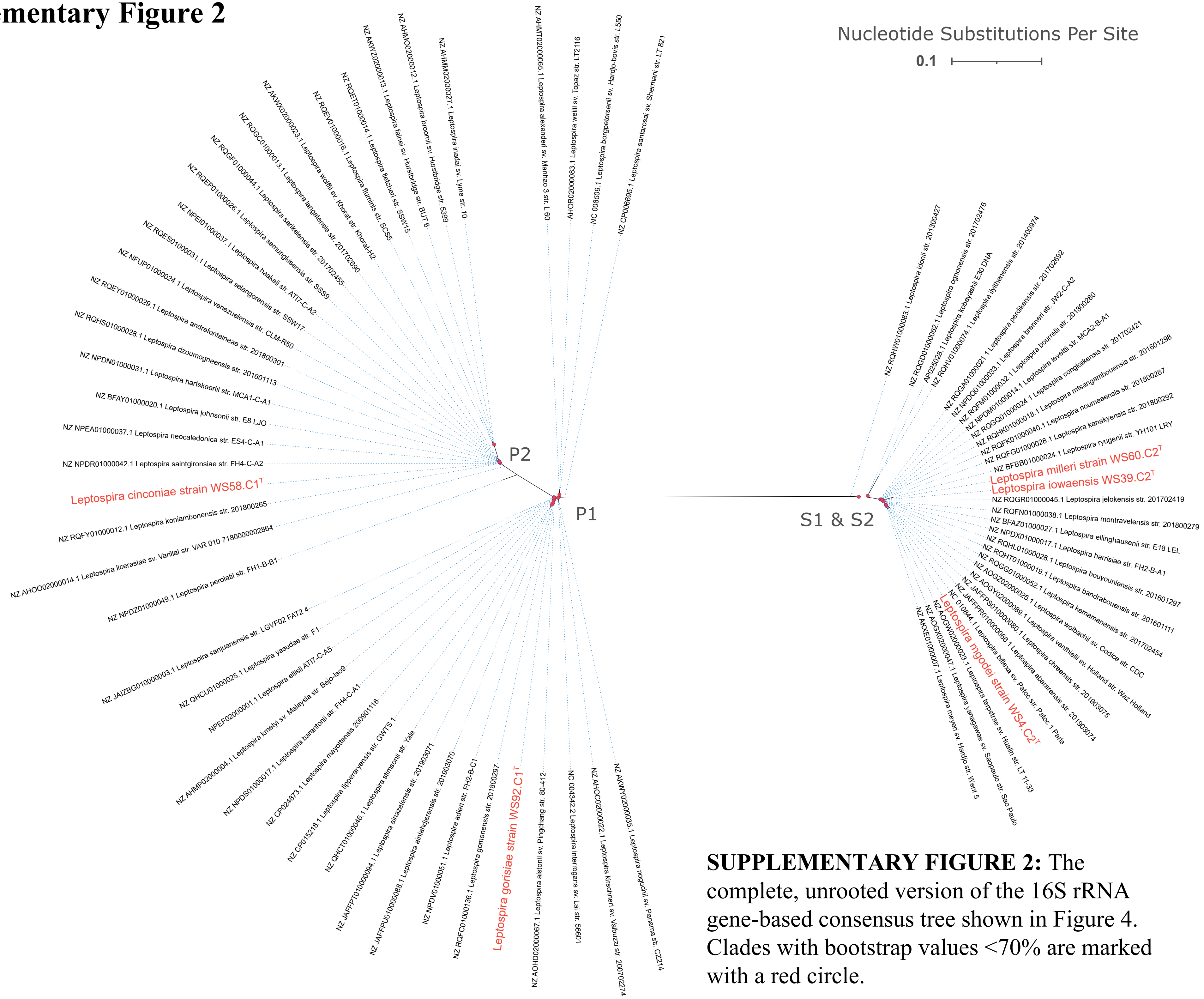

SUPPLEMENTARY FIGURE 2: The complete, unrooted version of the 16S rRNA gene-based consensus tree shown in Figure 4. Clades with bootstrap values <70% are marked with a red circle.

Supplementary Figure 3

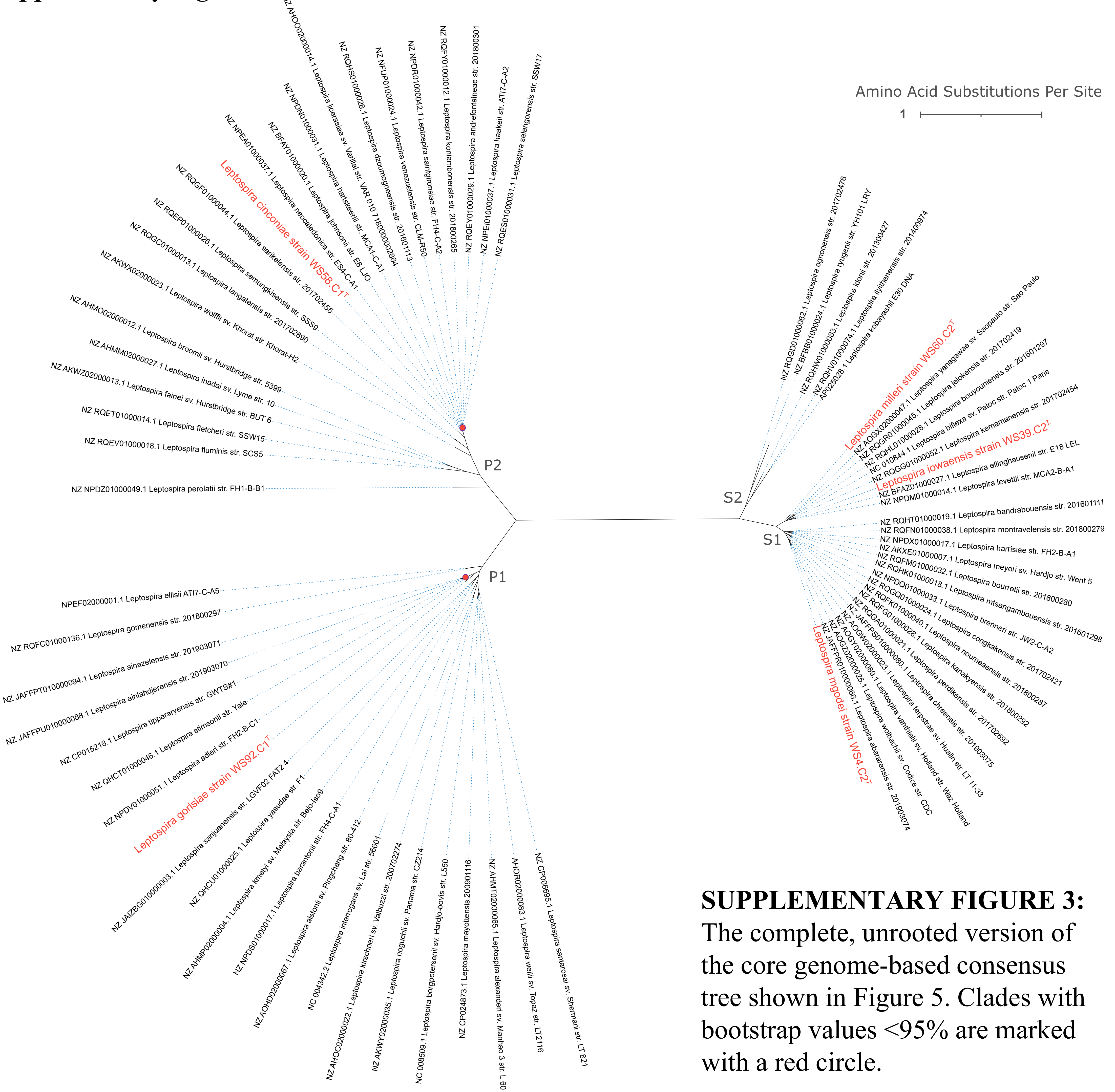

SUPPLEMENTARY FIGURE 3: The complete, unrooted version of the core genome-based consensus tree shown in Figure 5. Clades with bootstrap values <95% are marked with a red circle.
